# Practical Notes and Formulæ

**Published:** 1887-03

**Authors:** 


### PRACTICAL NOTES AND FORMULÆ.

**Hemorrhoids.**—The galvano cautery has been recommended as superior to all other methods for reducing the tumors, there is little pain and no hemorrhage, and the abraded surface readily heals.

**Tuberculosis.**—A writer (in *Medical World*) recommends the following as a superior remedy in tuberculosis. It relieves the cough and tends to fatten the patient:

℞. Syrup iodide lime.
Fl. extract malt, aa.............................℥iij
Fl. extract of hops..............................℥ij

Mix.—Teaspoonful three to four times a day.

**Fever Mixture.**—Dr. Peyre Porcher (in *New Orleans Medical Journal*) says of typhoid fever, after the use of cold sponging, etc., that the next most important auxiliary, and one that he regards as essential in every form of fever, is what he calls the "fever mixture," which is composed as follows, though the different ingredients may be varied to suit the case:

℞. Spts. etheris nitrosi...........................℥ss

Potass. acetatis.................................ʒi-ij

Potass. chloratis................................ʒj

Liq ammon. acetatis..............................℥j

Tr. aconiti......................................ʒss

Tr. opii camph...................................ʒij iij

Aquæ, q. s. ad...................................℥iv

M. Sig.—Dessertspoonful every two or three hours as long as there is fever.

Potassium bromide or morphia may be added if there is great restlessness and insomnia.

When the latter stage of the disease is complicated with severe broncho-pneumonia, the following formula has given satisfactory results:

℞. Vin. ipecac.....................................ʒj

Ammonii carb.....................................ʒij

Ammonii chloridi.................................ʒiij

Syr. simplicis...................................℥j

Aquæ, q. s. ad...................................℥vj

M. Sig.—Dessertspoonful every two hours in a wineglassful of water.

For the albuminuria which sometimes occurs, he gives three times a day two grains each of gallic acid and quinine.

For nausea or vomiting he finds most efficient drop-doses of wine of ipecac frequently repeated, or the following:

℞. Acidi carbolici..... ................................gtt.j,
Glycerinæ........................................ʒj,
Tr. opii camph.............................
Ess. menth. pip ...................................
Chloriformi pur. aa.................................gtt.v.

M. Sig.—In mucilag. acaciæ q. s. and repeat.

**"Restorers."**—This is a name applied to a class of compounds widely sold under the various names of "Hair Vigor," "Hair Renewer," etc. They came into notice first just after the Mexican war, under the name of General Twigg's Hair Dye. *All of these mixtures are poisonous.* A sample analyzed by the author showed the following formula, which does not differ materially from other compounds of the same class:

Lead acetate (sugar of lead)......8 grammes,

Milk of sulphur..................11 grammes,

Glycerine........................100 cubic centimeters,

Water............................½ liter.

**Ointment for Glandular Affections:**

℞. Ext. belladonna.......... ........................ʒj,

Ung. hydrargyri......................................ʒiv.

Dr. Kæmmerer asserts that the judicious use of this ointment, when used in time, will prevent suppuration, cause the enlargement or swelling to disappear, and the gland to resume normal action. Indicated in strumous and syphilitic affections.—*American Medical Journal.*

**To Clean Tarter from Teeth:**

℞. Dry hypochlorite of lime...........................ʒss,

Red coral.........................................ʒij.

M. S.—Triturate well and mix thoroughly. To employ it, moisten a new brush slightly, dip it into the powder, and apply to the teeth. A few days' use will produce a marked alteration in the appearance of the teeth.—*Scientific American.*

**Carbolic Acid in Felons.**—Dip the finger in carbolic acid and after waiting for half an hour prior to lancing, the cutting will be almost destitute of pain, much to the astonishment of the patient.—*Ex.*

**Cough Mixture.**—The following cough mixture is highly recommended by the editor of the *New England Medical Monthly*:

℞. Maltine............................................℥ij,
Glycerine.........................................℥j,
Syrup tolu.......................................
Syrup prun. virg. aa..............................℥v,
Syrup ipecac.......................................ʒv,
Syrup scillæ ......................................℥v,
Morph. sulph.......................................gr i,
Ammon mur.........................................ʒijss.

M.—Dessertspoonful every three or four hours.

For the relief of violent pains, that in some women precede the menstrual flow, Dr. Meniere, of Paris, gives a warm water enema, containing thirty grains of chloral and thirty grains of bromide of potassium. For young women only half of the above quantities should be prescribed.—*Rivista Balear.*

**Painful Dentition** :

℞. Cocaine hydrochlorate
Sodium borate aa.................gr.iv,
Syrup of althea.....................m.lxiv,
Syrup of poppy....................q. s. to make ʒii.

M —A little to be rubbed on the gums several times a day.—*L' Union Medicale.*

**Treatment of the Cystitis of Blennorrhagia.**—Take every day three of the following powders in an infusion of flaxseed :

℞. Powdered leaves of hyosciamus................gr.xx,
Sugar..................................dr.iv.

Divide into seven powders. If the pain resists, a tablespoonful, every half hour, of the following infusion will be found very efficacious :

℞. Hyosciamus leaves............................gr.xlv,
Boiling water...............................oz.iv.

If dryness of the throat is felt or somnolency, the administration should cease and a cup of strong coffee will set all right. By these means, and in a few hours the most severe pain is eased.—*Medical Press and Circular.*

**Indigestion.**—Dr. C. M. Dickson (in *The Medical World*) writes : Now, doctor, I am going to give you a "*pointer*," and if you follow it *honestly*, you will want no better sport, than to go about the country *knocking the conceit out of all cases of indigestion* which fall into your hands.

In the first place I would have you give to each patient who complains of a sense of weight, or soreness in the *pit* of the stomach, the following cathartic :

℞. Hydrarg. chlor. mitis...........................gr.j,
Sacchari alb.... ...........................gr.v.

M. Ft. chart, No. 1.

Sig.—Take at bedtime in a tablespoonful of milk or water.

After this has operated, put the patient upon the following, and continue it for at least *two months:*

℞. Tr. nucis vomicæ............................ ...

Acid. salicylici..................................

Potassæ bicarb aa............................ʒij

Aquae dist., q. s. ad.............................f℥iv

Sig.—A teaspoonful in a wineglass of water one-half hour before meals.

This preparation should be wrapped in some colored paper to keep out the light, and kept in a cool place.

If at any time the bowels become constipated, relieve them by giving the cathartic mentioned above.

